# Epidemiological survey on the practice of allergen immunotherapy in Brazil

**DOI:** 10.1186/1939-4551-8-S1-A189

**Published:** 2015-04-08

**Authors:** José Luiz De Magalhães Rios, Norma Rubini, Andrea Cohon, Martti Anton Antila, Veridiana Pereira-Aun, Rosa Cristina Oliveira Gaia Duarte, L Karla Arruda, Fabio Fernandes Morato Castro

**Affiliations:** 1 Brazilian Association of Allergy and Immunology,Brazil; 2 Policlínica Geral Do Rio De Janeiro, Brazil; 3 Federal University of the State of Rio De Janeiro, Brazil; 4 Hospital Infantil Darcy Vargas, Brazil; 5 Hospital Servidor Público Estadual De São Paulo, Brazil; 6 Ribeirao Preto Medical School, University of Sao Paulo, Brazil; 7 University of São Paulo, Brazil

## Background

Allergen immunotherapy (IT) is widely used to treat patients with allergic diseases, proven effective and recommended by national and international guidelines. Its efficacy and safety depend on the correct indication, use of relevant allergens and good quality extracts, as well as prescription and supervision by a trained professional. Our aims were to evaluate the frequency of use of IT in Brazil and to investigate whether this practice meets recommendations of the Brazilian Association of Allergy and Immunology (ASBAI) and Brazilian Medical Association.

## Methods

Standardized questionnaire consisting of 20 items was applied to physicians participating in the annual congress of ASBAI. The questionnaire was anonymous and voluntary, and evaluated data related to medical training, board certification, duration of professional practice, indications for IT, age limitation, mode of administration and schedules, type and standardization of extracts, duration of IT and administration site.

## Results

Four-hundred twenty two questionnaires were completed; 88.2% of participating professionals reported training in Allergy and Immunology, and 57.8% were Board certified in Allergy and Immunology. Sixty-eight percent practice the specialty >5 years; 79.2% work in private practice and 80.6% perform IT in clinical practice. Among IT prescribers, 121 (35.6%) are not Board certified by ASBAI. IT is used most often with the following indications: rhinoconjunctivitis - 97.9%, cutaneous reactions to mosquito bites - 75.1%, asthma - 72.2%, atopic dermatitis - 57.4%, allergy to insect stings - 52.1% and food allergy - 7.9%. The data revealed that 75.6% of professionals prescribe IT for children <5 years and 48.2% for patients> 65 years. Mode of IT included SCIT - 91.7%; SLIT - 52.9%; SCIT rush - 7.1% and oral desensitization - 10.9%. The duration of IT was: <3 years - 38.7%, 3-5 years - 58% and> 5 years - 1.2%. IT is conducted in health care units in 88.1% of cases.

## Conclusions

Both SCIT and SLIT are widely used in Brazil. Most professionals who use IT have training in Allergy and Immunology (88.2%), however, only 57.8% are Board certified. The practice of IT occurs predominantly in private clinics. A significant percentage of physicians perform practices which are not in full agreement with national and international recommendations, including prescription of IT for children <5 years and patients > 65 years, and duration of IT less than 3 years in 38.7% of cases.

